# Supplementary material for: Influenza A Virus Infection Reactivates Human Endogenous Retroviruses Associated with Modulation of Antiviral Immunity
Source: Viruses. 2022 Jul 21;14(7):1591. doi: 10.3390/v14071591 (PMC9320126; doi:10.3390/v14071591)
Supplement: Supplementary file 1 [file viruses-14-01591-s001.zip › MotifAnalysis.zip!/MotifOutput_SC35MdNS1/homerResults/motif4.similar.html]

motif4

## Information for motif4

G
A
T
C
C
G
T
A
C
T
G
A
A
C
T
G
C
T
A
G
C
G
A
T
A
G
C
T
A
C
G
T
A
C
T
G
A
C
G
T
C
G
T
A
C
G
T
A
  
Reverse Opposite:  

C
G
A
T
G
C
A
T
C
G
T
A
A
G
T
C
C
G
T
A
C
T
G
A
C
G
T
A
A
G
T
C
A
G
T
C
A
G
C
T
A
C
G
T
C
T
A
G
  

|  |  |
| --- | --- |
| p-value: | 1e-68 |
| log p-value: | -1.588e+02 |
| Information Content per bp: | 1.810 |
| Number of Target Sequences with motif | 40.0 |
| Percentage of Target Sequences with motif | 51.95% |
| Number of Background Sequences with motif | 240.1 |
| Percentage of Background Sequences with motif | 0.53% |
| Average Position of motif in Targets | 96.1 +/- 53.3bp |
| Average Position of motif in Background | 86.0 +/- 96.5bp |
| Strand Bias (log2 ratio + to - strand density) | 5.4 |
| Multiplicity (# of sites on avg that occur together) | 1.34 |
| Motif File: | file (matrix) reverse opposite |

### Similar de novo motifs found

|  |  |  |  |  |  |  |  |
| --- | --- | --- | --- | --- | --- | --- | --- |
| Rank | Match Score | Redundant Motif | P-value | log P-value | % of Targets | % of Background | Motif file |
| 1 | 0.865 | C T A G A C T G A C T G C G A T A C G T A C G T A C T G A C G T C T G A C G T A | 1e-68 | -156.632997 | 48.05% | 0.37% | motif file (matrix) |
| 2 | 0.902 | A G T C C G T A C G T A A C T G A C T G A C G T A C G T A C G T A C T G A C G T | 1e-65 | -151.607722 | 42.86% | 0.22% | motif file (matrix) |
| 3 | 0.780 | A C T G A C T G C G T A A C G T A C G T A C T G A C G T C G T A | 1e-58 | -134.125297 | 48.05% | 0.68% | motif file (matrix) |
| 4 | 0.700 | C T A G A C T G A C T G A C G T A C G T A G T C A C T G A C G T | 1e-37 | -86.927467 | 48.05% | 2.48% | motif file (matrix) |
| 5 | 0.729 | C G A T A C G T C T G A A G T C C G T A C G T A A C G T G T A C A G T C A C G T | 1e-16 | -38.867367 | 15.58% | 0.30% | motif file (matrix) |
